# A Rare Case of a Lost Suture Needle during Third Molar Surgery

**DOI:** 10.1155/2015/372153

**Published:** 2015-08-12

**Authors:** Sertac Aktop, Gokhan Gocmen, M. Elif Özturk, Onur Gonul, Altan Varol

**Affiliations:** Department of Oral and Maxillofacial Surgery, Faculty of Dentistry, Marmara University, 34365 Istanbul, Turkey

## Abstract

The authors report a case that is started with a simple upper third molar's surgical extraction and a broken 3.0 suture needle tip incident occurred. Broken fragment's localization has been detected with 3D cone beam computed tomography (CBCT). Needle tip has been reached with the help of CBCT vision. CBCT's benefits have been discussed on these types of cases and further migration of the needle tip during surgical procedure is reported.

## 1. Introductıon

Accidental breakage of suture or injection needles during oral surgical procedures is a rare but serious complication. It commonly occurs due to excessive force applied on the tip or sudden movement of the patient, especially if their mechanical properties are weak [1]. Finding the location of the broken fragment and its potential migration to deeper layers are the issues that make this complication challenging. Sometimes further procedures and radiological examinations might be necessary to overcome this condition [2].

Herein, we report a case that is started with a simple upper third molar's surgical extraction and required further interventions to find out an accidentally broken 3-0 suture tip. A lost broken suture needle was located using cone beam computed tomography scan and retrieved via surgery.

## 2. Case Presentation 

27-year-old male patient referred to our clinic complaining about his right upper third molar. According to clinical and radiographical examination, moderate infection was inspected, caused by wisdom molar. In order to cure infection and its symptoms the tooth was decided to be extracted. Suture needle was found to be broken while passing through the palatinal mucosa. Although it was tried to localise the broken needle fragment, it could not be managed to reach it in deep mucosal area. The broken fragment could not be prevented from migrating to deeper tissues while attempting over reaching. The flap was closed and the patient was gone under further diagnostic examination.

**Figure 1:**
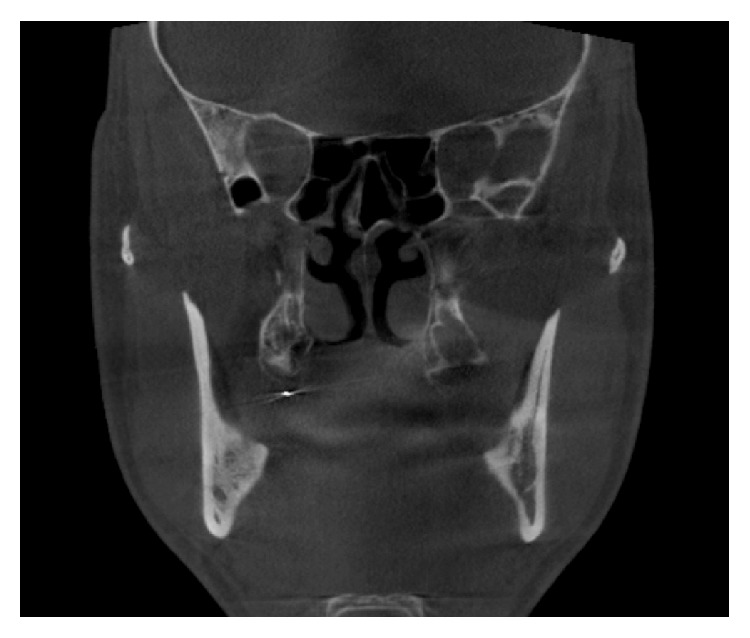
Coronal view.

**Figure 2:**
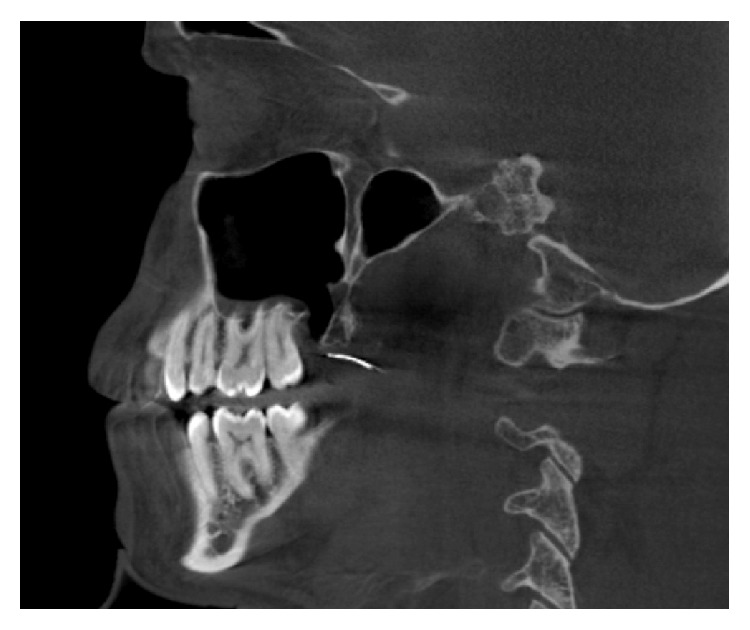
Sagittal view.

**Figure 3:**
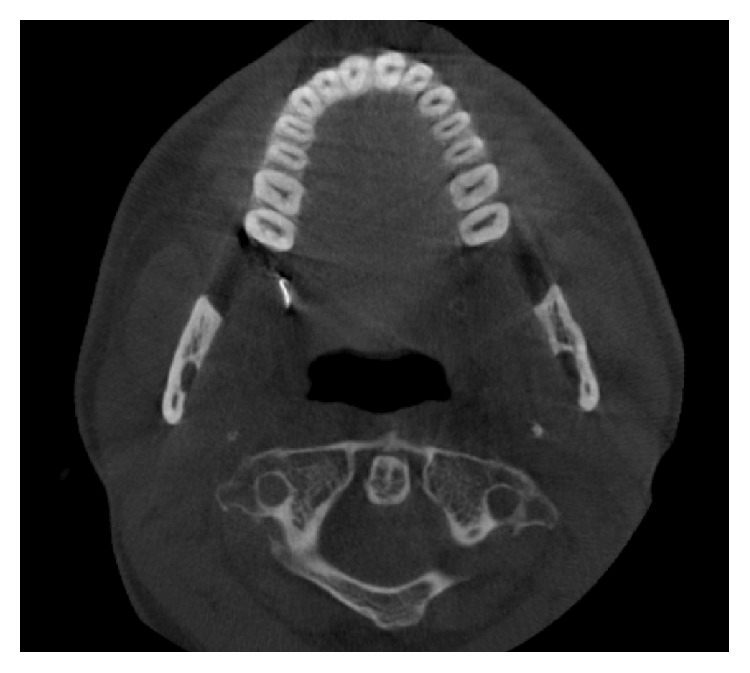
Axial view.

3D cone beam computed tomography (CBCT) (ProMax 3D Mid Machine, Planmeca Oy, Helsinki, Finland) and conventional OPTG were performed to localize the needle. According to tomographic images, location of needle was about under the palatinal mucosa, the region of right maxillary tuberosity, nearly just below the medial pterygoid plate. The broken fragment was detected distant from the localization it had been broken at the first place (Figures 1, 2, and 3).

**Figure 4:**
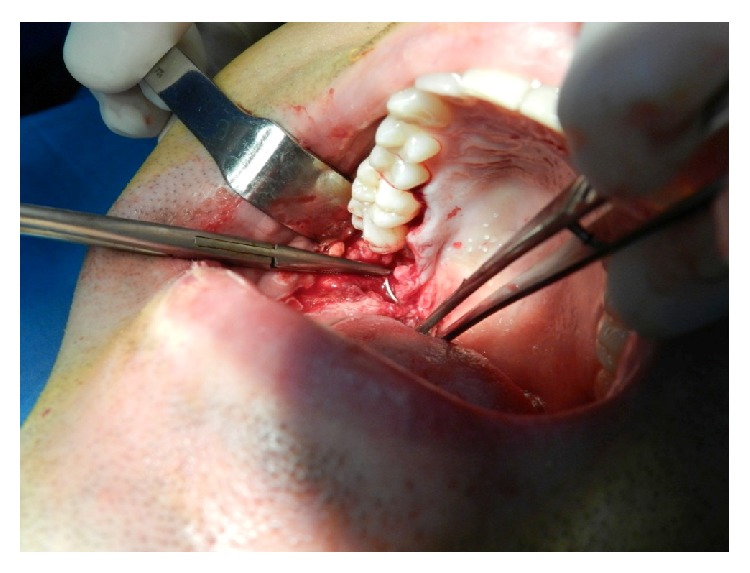
Intraoperative view.

After detecting the exact location of the needle, the patient has gone under a second surgery immediately and the needle was reached with a more extensive incision of 35 mm from former horizontal incision of third molar surgery through buccal mucosae inferiorly. Blunt dissection was made through oropharyngeal region and the broken fragment was removed successfully (Figure 4).

## 3. Discussion

A broken suture needle may be clinically visible or fully embedded after breakage. Visible fragments may be retrieved immediately using a hemostat [3]. Several methods were described to determine the position and removal of dislodged foreign bodies in maxillofacial region [4]. Cohen reported a case of a removal of an imbedded broken suture needle by a simple magnet [5]. Also removals of the broken needles in maxillofacial region using fluoroscopy were also reported [2]. However, if the fragment is not visible in the oral cavity, attempts to palpate the mucosa to locate it may drive it deeper and complicate retrieval [1], as we have experienced after taking CBCT. As some authors have suggested that removal should be indicated only for symptomatic reasons, others support immediate intervention. The risk of infection or migration or the presence of symptoms such as pain and trismus necessitates the retrieval of the retained or broken needles from maxillofacial region [6]. The timing of the retrieval surgery is also another issue. Some authors have advocated early removal to minimize migration and patient anxiety, while others believe that retrieval can be postponed for two to four weeks in asymptomatic patients, to allow a fibrous capsule to form [1]. Hassani et al. mentions a case of a broken needle intervention, in which the broken needle remained two years without any symptom. In this case [1], the decisive point which make us remove the needle rapidly was the anxiety of patient. Then, we decided to remove the needle just after the CT aided localization in order to prevent excessive mobilization of the needle while waiting for capsulation. But also, we have experienced the uncontrolled migration of the broken fragment to deeper tissues by indirect pushing forces while dissecting the surrounding tissues as we searched for the missing fragment.

As the CT scanning is considered an accurate method of imaging that indicates the position of a needle in relation to recognizable anatomical landmarks [2] and gives the coordinates of the foreign body for the surgical intervention, possible migration of the foreign body during the surgery could make the situation challenging. Particular measurements on CBCT scans could be useless when the work is put into practice. Any radiography technique, like C-arm fluoroscopy, that would allow chasing the missing fragment during the surgery could be helpful and practical.

## 4. Conclusions

CBCT can be able to detect the fragment accurately, leading to a more specific surgical procedure and decreasing operating time but handling the surgical procedure gently is of great importance. Surgical access may be limited by the presence of vital structures like nerves and arteries. Otherwise, the procedure might get more complicated by the migration of the broken fragment to deeper portions of oropharyngeal tissues.
